# Are arthropods at the heart of virus evolution?

**DOI:** 10.7554/eLife.06837

**Published:** 2015-03-09

**Authors:** Gytis Dudas, Darren J Obbard

**Affiliations:** Institute of Evolutionary Biology, University of Edinburgh, Edinburgh, United Kingdom; Institute of Evolutionary Biology and Centre for Immunity, Infection and Evolution, University of Edinburgh, Edinburgh, United Kingdomdarren.obbard@ed.ac.uk

**Keywords:** RNA virus, evolution, arthropods, segmentation, negative-sense, phylogeny, viruses

## Abstract

The huge diversity of negative-sense RNA viruses in insects, spiders and other arthropods suggests that these animals could be central to virus origin and evolution.

**Related research article** Li CX, Shi M, Tian JH, Lin XD, Kang YJ, Chen LJ, Qin XC, Xu J, Holmes EC, Zhang YZ. 2015. Unprecedented genomic diversity of RNA viruses in arthropods reveals the ancestry of negative-sense RNA viruses. *eLife* **4**:e05378. doi: 10.7554/eLife.05378

**Image** Arthropods are associated with a wide variety of negative-sense RNA viruses (black)

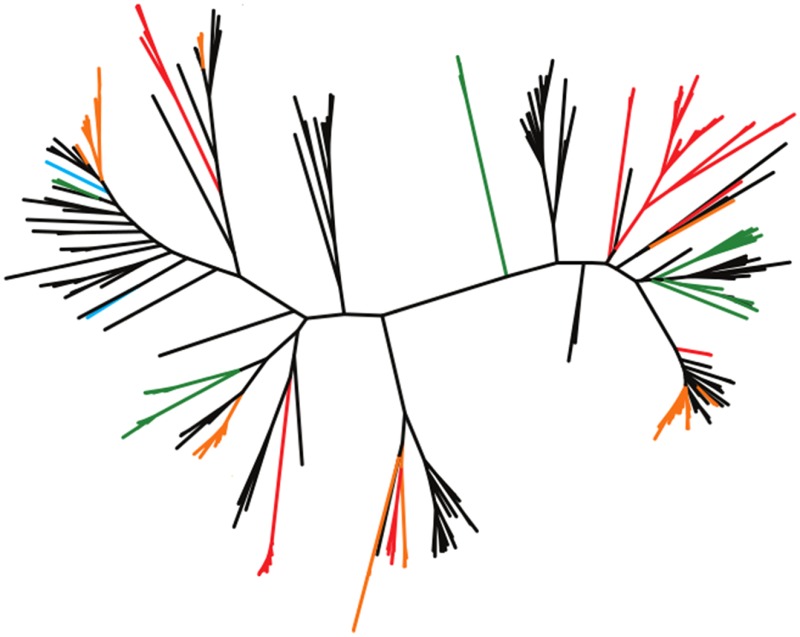

Viruses are the most numerous, and arguably the most diverse, branch of life (Koonin and Dolja, 2013). They have been described as the ‘dark matter’ of biology because they can be very hard to detect, and the large-scale sequencing of genetic material from the environment is only now showing us how numerous and diverse they really are. These ‘metagenomic’ sequencing studies have revolutionized our knowledge of viruses in many settings, including in saltwater and the faeces of vertebrate animals (e.g., Delwart, 2007; Kristensen et al. 2010; Rosario and Breitbart, 2011; Willner and Hugenholtz, 2013).

However, most of these analyses have focused on viruses with genomes that are made of DNA or positive-sense RNA, and there have been fewer studies of viruses with genomes made of negative-sense RNA. Therefore, the evolutionary history of negative-sense RNA viruses, which are responsible for influenza, measles, Ebola and many other diseases in animals and plants, remains obscure. Now, in eLife, Yong-Zhen Zhang—who is based at the Chinese Center for Disease Control and Prevention—and co-workers have taken a new approach for studying the diversity of viruses. By performing metagenomic sequencing on a diverse collection of insects, spiders and other arthropods, they have uncovered a previously unsuspected depth and breadth to the negative-sense RNA viruses (Li et al. 2015).

Zhang and co-workers—who are also based at the University of Sydney and Centers for Disease Control and Prevention in Wuhan and Wenzhou—sequenced all of the RNA extracted from 70 arthropod species collected across China. Within this RNA they uncovered the genomes of 112 new negative-sense RNA viruses, and they inferred the evolutionary relationships between the viruses using phylogenetic trees based on the RNA polymerase gene. Zhang and co-workers found that the 112 new viruses were spread across the major lineages of the negative-sense RNA viruses (Figure 1; Li et al. 2015). These discoveries fill some major gaps in our knowledge, and allow the tree of viral relationships to be updated. For example, this latest work confirms that the viruses of the Arenaviridae genus—which generally infect rodents—belong to the Bunyaviridae family along with two previously unclassified genera of viruses that infect plants (Ecker et al. 2005; Kormelink et al. 2011).

**Figure 1.**
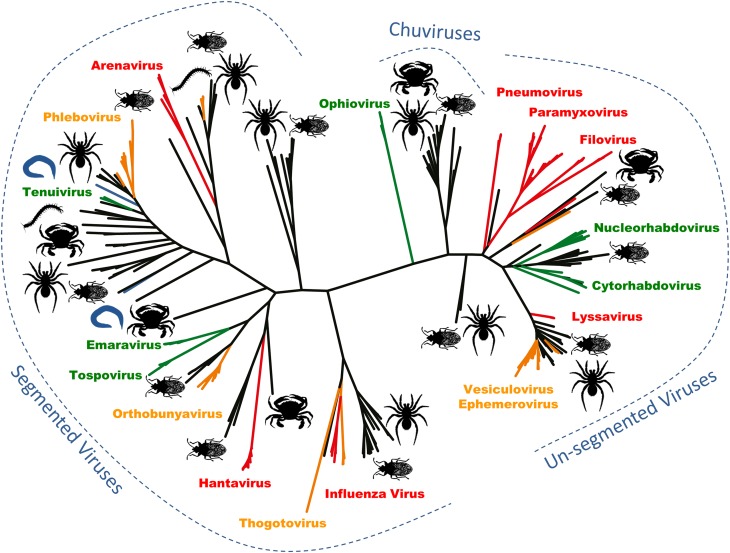
Arthropods are hosts to most of the major groups of negative-sense RNA viruses. A phylogenetic tree—adapted from Li et al.—that infers the evolutionary relationships between all the groups of negative-sense RNA viruses, based on the sequence of the RNA-dependent RNA polymerase gene. Viruses that infect vertebrates are colored red if they are transmitted directly between vertebrates and orange if they are carried by arthropods. Viruses that infect plants (including those carried by arthropods) are shown in green, and viruses that infect nematodes and flatworms are shown in blue. Zhang and co-workers discovered many of the other viruses, colored in black, in association with insects, crustaceans, spiders and other arthropods. The large number and wide distribution of the arthropod-associated viruses suggests that arthropods could be central to the evolution of these viruses, although better sampling of other invertebrates (such as nematode worms) would help to confirm this. The newly-identified Chuviruses are found between the segmented and unsegmented viruses.

They also identified a completely new virus lineage that they named the Chuviruses (Figure 1). The genomes of some members of this lineage are formed from a single piece of RNA, while the genomes of others are ‘segmented’ into multiple pieces of RNA. Therefore, the Chuviruses seem to provide an evolutionary link between the lineages of non-segmented and segmented viruses. In addition to illustrating the rapid pace of change in our understanding of the diversity of viruses, these findings also highlight a potential need to overhaul the way in which we catalogue and classify viral genome sequences (e.g., Brister et al. 2015).

Even though this deep phylogenetic analysis is extremely challenging—and should be treated as tentative—the sheer number and diversity of these new viruses suggest that the arthropods may have been important in viral evolution. Indeed, Zhang and co-workers argue that the origin and diversification of these viruses could be directly linked to arthropod biology. In particular, the unparalleled numbers and diversity of the arthropods, combined with the wealth of parasitic lifestyles that arthropod species display, may mean these animals are uniquely placed as hosts and carriers of viruses to act as a hotspot of viral evolution. This is an appealing hypothesis, and one that now warrants considerable attention.

Nevertheless, a few important questions remain. First, although the virus genome sequences were found in the arthropod samples, it remains uncertain that these sequences represent active viral infections of these animals. Instead, it is possible that the genome sequences may come from viruses that are associated with organisms the arthropods eat, or parasites they carry (such as nematode worms or single-celled eukaryotes). Experiments that confirm whether the arthropods are the hosts of these viruses are likely to follow soon, but at present the jury is still out.

Second, although a surprising number of negative-sense RNA viruses were found in arthropods, the study by Zhang and co-workers is the first such large-scale survey of this type. This may bias our perspective, and an equivalent survey of a different clade of animals could revolutionize our understanding of these viruses yet again. Although there have been substantial surveys of viruses associated with terrestrial vertebrates, there are relatively few species (∼30,000), so this may limit the diversity of the viruses present in these animals. The nematodes, which are more numerous than the arthropods and might rival them for diversity (Platt, 1994), could be especially illuminating. Zhang and co-workers note that some of the viruses present in the arthropod samples are related to viruses found in studies of nematodes and flatworms. Thus, until more surveys have been done, it will be hard to be certain that the arthropods are special.

This study also raises some exciting new questions. For example, while many of the negative-sense RNA virus lineages were found in the arthropods, some were striking in their absence (Li et al. 2015). Amongst the virus family Orthomyxoviridae, for example, they identified many relatives of the Quaranja viruses, which infect ticks, but none that are closely related to the Influenza viruses that infect vertebrates. Similarly, no lineages were found to be closely related to Ebola and other Filoviruses, which belong to a lineage that apparently only infects mammals (Ecker et al. 2005). It is therefore interesting to speculate that these virus groups may genuinely display some long–term association with mammals.

Finally, although the approach used by Zhang and co-workers should work equally well for all classes of virus, the present study only reports on data from those with negative-sense RNA genomes. Therefore, even though the other groups of viruses have been sampled more broadly in the past, we still expect to see some more surprises emerge from this unprecedented dataset.
